# Atraumatic Acromioclavicular Dislocation: A Case Report and Review of the Literature

**DOI:** 10.1155/2017/8450538

**Published:** 2017-04-05

**Authors:** Nasrat Sadeghi, Pieter Stijn Haen, Ron Onstenk

**Affiliations:** Groene Hart Ziekenhuis, Bleulandweg 10, 2803 HH Gouda, Netherlands

## Abstract

Acromioclavicular dislocation (AC dislocation) is a common injury of the shoulder. In contrast to a traumatic cause, nontraumatic dislocation is very rare. We report on a 17-year-old female that presented with voluntary recurrent dislocation of the right AC joint followed by recurrent pain without instability of the ipsilateral shoulder. Clinical examination showed crepitation as well as palpitation pain and dislocation of the AC joint. There were no symptoms of Marfan or Ehlers-Danlos syndrome as other joint examinations were also negative for hypermobility. Considering age as well as minor complaints, nonoperative treatment by postural therapy without taping was recommended. After one year, the patient experienced fewer symptoms and she was able to participate in daily activities.

## 1. Introduction

Acromioclavicular dislocation (AC dislocation) is a common traumatic injury of the shoulder. Sprain and dislocation are the most common diagnoses after trauma of the shoulder. Males are more affected than females with a male-to-female ratio of 5 : 1 and an incidence peak between the ages of 20 and 30 [1]. AC dislocation is often caused by direct trauma, mostly falling on the affected side. In contrast to voluntary dislocation of the glenohumeral joint, a voluntary dislocation of the AC joint is rare [2–4].

The aim of this paper is to report a case of voluntary dislocation of the right AC joint in a 17-year-old female. In addition, the literature of atraumatic dislocation of the AC joint will be discussed.

## 2. Case

A 17-year-old Caucasian female presented with progressive pain and instability of her right shoulder at our outpatient clinic. During the last couple of years, she had noticed pain with a recurrent click in the front of her right shoulder after elevation over 90 degrees in combination with forward movement of her arm. Precedent trauma of the shoulder was denied and she had no additional complaints of other joints.

External rotation of both shoulders amounted to 90 degrees with a full range of motion in the anterior and abduction plane. Glenohumeral instability tests as apprehension, relocation, and hyperabduction as well as sulcus sign were all negative for both sides. Therefore, major instability or laxity of the right glenohumeral joint could not be determined. Notable were crepitation, laxity, and local pressure pain of the right AC joint. Crossover was not painful. The patient could actively provoke a dislocation of her right AC joint by abduction and external rotation of her shoulder. This could be also voluntarily reduced, but accompanied by pain. All other specific tests like Adson test were negative. Examination of the left shoulder and other joints showed no abnormalities. There were no signs or symptoms of Marfan or Ehlers-Danlos syndrome. Beighton score was only positive in forward flexion of the trunk, which means a general joint laxity was not present.

**Figure 1:**
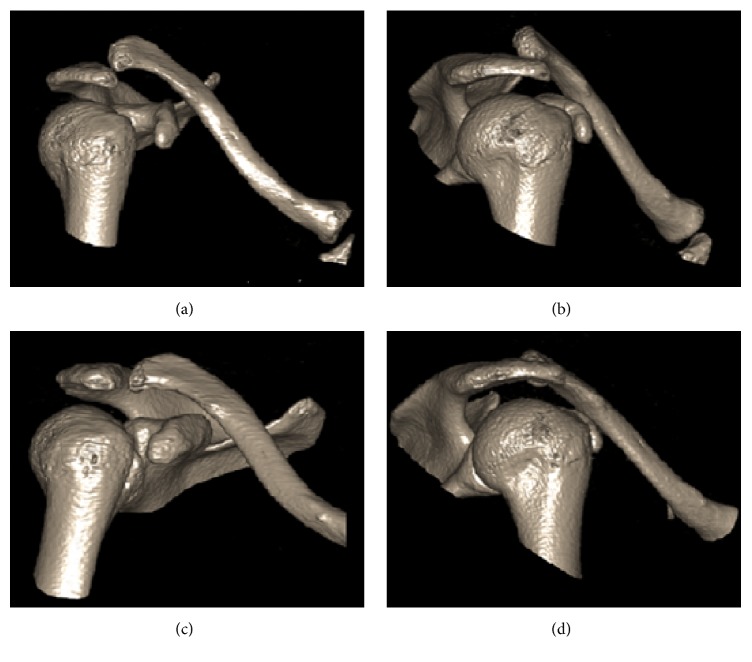
3D-CT scan after the patient created a dislocation ((a) and (b)) and after the patient created a comfortable situation ((c) and (d)).

X-ray studies and MRI-arthrogram of the right shoulder and clavicle showed no abnormalities of labrum, ligaments, and acromioclavicular joint. In particular, the coracoclavicular ligament (CC ligament) showed no abnormalities. 3D-CT reconstruction was performed during the manoeuvre in which the patient creates a dislocation of the AC joint (Figure 1). Figures 1(a) and 1(b) show a significant change (shaft length) in relation between clavicle and acromion. Figures 1(c) and 1(d) show a situation in which the patient created a comfortable situation after voluntary reduction. These figures show a normal anatomic position of the AC joint and not a dislocation anymore.

The diagnosis was defined as voluntary dislocation of the acromioclavicular joint without major glenohumeral instability or other generalized joint abnormalities.

## 3. Discussion

Only four cases with this condition have been published. In 1977, Janecki Jr. [5] published a case of a 19-year-old female patient with bilateral voluntary dislocation of both AC joints. This patient had no history of trauma. She could actively dislocate both AC joints anteriorly by retraction of her scapula. By protraction of her scapula, she could reduce the dislocation. Both manoeuvres were associated with pain. Treatment consisted of detailed explanation of the problem including advice to avoid manoeuvres causing dislocation or pain. After one month, the patient was asymptomatic.

The second case was published by Sahara et al. [6] in 2005. A 19-year-old Japanese male presented with persistent pain with a recurrent click of his right shoulder with no history of trauma. X-ray showed posterior dislocation of both AC joints. CT scan and MRI revealed no abnormalities. Operative stabilization of the AC joint was performed. The coracoid tip with conjoined tendon was osteotomized at 1.5 cm from the tip after drilling the tip longitudinally. A trough to receive the tip was made in the anterior aspect of the clavicle around the attachment of the trapezoid ligament, and the osteotomized coracoid tip with conjoined tendon was then transferred and fixed to the trough with a screw and a washer. The distal end of the clavicle was reduced anteriorly and did not touch the acromion. Two Kirschner wires were inserted from the lateral acromion into the distal end of the clavicle. Twenty-two months after the surgery, the pain was reduced, but a slight click still remained around the AC joint during arm elevation. Posterior displacement, which was 18 mm preoperatively on the axillary view of the X-ray, was reduced to 8 mm in 5 months after surgery. However, 22 months after surgery, posterior displacement increased to 15 mm.

Richards et al. [7] reported a case of voluntary AC dislocation in a 14-year-old boy in 1986. This patient suffered from spastic hemiparesis. An earlier trauma could not be ruled out. Since the patient was asymptomatic, no treatment was indicated.

The exact aetiology of this condition stays unclear. The patients, mentioned above, are treated in different ways. Therefore, more experience is needed before we could design a protocol for managing this condition. It is remarkable that in all the cases the symptoms started around puberty. This could be a result of underdevelopment of the ligaments, even though MRI shows no abnormalities of the ligaments. More research is needed to understand the role of CC ligament.

Considering age as well as the present minor complaints in our patient, nonoperative treatment was recommended by detailed explanation including refrain from specific manoeuvres (active abduction in combination with external rotation) that provokes dislocation as well as postural therapy without taping. After one year, the patient could still provoke AC dislocation. However, she experiences fewer symptoms and is able to participate in daily activities. It can be concluded that the patient adapted to this condition. Avoiding voluntary AC dislocation protects her from pain and inflammation. Operative AC stabilization in a way as performed in AC traumatic dislocation should be considered in case of therapy-resistant symptoms.
